# Supplementary material for: Repurposing FIB-4 index as a predictor of mortality in patients with hematological malignancies and COVID-19
Source: PLoS One. 2021 Sep 23;16(9):e0257775. doi: 10.1371/journal.pone.0257775 (PMC8459998; doi:10.1371/journal.pone.0257775)
Supplement: S1 File — (DOCX) [file pone.0257775.s001.docx]

**Highlights**

FIB-4 index >3.85 was independent predictor of mortality in COVID-19 with hematological malignancies

FIB-4 index >3.85 has 79% sensitivity and 84% specificity for predicting mortality

FIB-4 >3.85 confers to 80% posterior probability of mortality and FIB-4 <3.85 to 19% probability.
