# Supplementary material for: Repurposing FIB-4 index as a predictor of mortality in patients with hematological malignancies and COVID-19
Source: PLoS One. 2021 Sep 23;16(9):e0257775. doi: 10.1371/journal.pone.0257775 (PMC8459998; doi:10.1371/journal.pone.0257775)
Supplement: Attachment — Submitted filename: Response to Reviewer 2.docx [file pone.0257775.s002.docx]

Reviewer #2: The authors addressed points that were raised by the reviewers adequately.

The only minor point is, in Page 8, "Consecutive sampling of adults who were tested positive for COVID19 while undergoing chemotherapy for hematological malignancy" would be a better description of the population analyzed.

Response: Thank you very much for the suggestion, we have revised accordingly.
